# Resilient health care performance in the real world: fixing problems that never happened

**DOI:** 10.1186/s12913-024-11639-z

**Published:** 2024-10-17

**Authors:** Jeffrey Braithwaite, Kate Churruca, Louise A. Ellis, Elle Leask, Janet C. Long, Mitchell Sarkies, Yvonne Zurynski, Robyn Clay-Williams

**Affiliations:** 1 Centre for Healthcare Resilience and Implementation Science, Faculty of Medicine, Health and Human Sciences, Australian Institute of Health Innovation, Macquarie University, Sydney, Australia; 2 International Society for Quality in Health Care (ISQua), Dublin, Ireland

**Keywords:** Complex systems, Resilient performance, Improvement, Intervention, Systems change, Sharp end behaviours

## Abstract

**Background:**

Staff in health systems everywhere have exhibited flexibility and a capacity for improvisations during, and in response to, the COVID-19 pandemic. Looking to other examples of such resilient behaviours outside of those induced by the pandemic is instructive for those involved with researching or understanding change, or making health systems improvements.

**Methods:**

Here, we synthesise and then assess the value of eight case studies of in situ resilient performance from Canada, Sweden, Japan, Belgium, the United Kingdom, Norway, the United States and Brazil. The cases are divided into four categories: responsiveness to a crisis; adaptiveness over time; local adoption in accommodating to a top down, national policy change; and the consequential outcomes of an intervention.

**Results:**

The cases illuminate the resourcefulness of translational and social researchers in examining such behaviours and practices. More than that, they also foreground the ingenuity and adaptive capacity of staff on-the-ground who continually anticipate, respond and adapt to make systems work and provide continuous care in the face of many challenges, including resource deficiencies, policy misalignments, and new technologies, policies and procedures that need to be integrated into local workflows. Front line clinicians make care systems work, pre-empting issues and sorting out problems before they occur or as they arise.

**Conclusions:**

A key lesson amongst a range of findings is that, rather than focusing on shiny new tools of change (checklists, frameworks, policy mandates), it is much more insightful and satisfying to deeply apprehend care at the sharp end, where clinicians deliver care to patients, understanding how everyday work is executed. This, rather than the Health Ministry, the Boardroom, or the Management Consultant’s office, is where and how change is being enabled, and where street level actors solve problems, thwart issues in advance, and constantly avoid pitfalls.

## Background

If the COVID-19 pandemic has taught us anything, it is the lesson that healthcare systems are, when push comes to shove, adaptive and flexible [1] —with performance we have described through the label *Resilient Health Care.* [2–9] Resilience, in this vein, is the capacity of a system to maintain its performance before, during and after some level or type of disturbance [2]. Seeing healthcare through this lens invites us to look at how everyday clinical work actually gets done, and to notice that the same interactions and behaviours of healthcare teams can result in variable outcomes (e.g., successes or failures), depending on a host of emergent and dynamic behaviours and circumstances [10]. In the context of COVID-19, elements of resilience that are associated with effective responses include: adapting; cutting through a plethora of change events and bureaucracy; building capacity while preserving functionality of the health system; manoeuvring to continue care in the light of initiatives such as lock-downs and rapidly changing policies; and reducing vulnerability to catastrophic losses in communities, while continuing to learn, monitor and adjust to an ongoing stream of dynamic situations [11]. 

That does not mean that healthcare is perfect; indeed, many health systems are said to be in crisis, e.g., Canada’s [12, 13], and the National Health Services of the UK [14, 15]. But front-line teams, navigating these pressures, nevertheless deliver excellent care, mainly due to the workforce’s capacities, training, expertise and commitment [9, 16, 17]. In a constant stream of behavioural repertoires, clinicians provide treatment and services including sorting out complications, taking pre-emptive action, and nipping problems in the bud. So: while there are fragilities, cracks and fissures in modern health systems some of which are attributable to lack of resources, poor information technology, burnout, excessive bureaucracy and other post-peak pandemic manifestations, many patients receive good outcomes from clinicians flexing and adjusting to these pressures, events and circumstances.

Proponents of Resilient Health Care have theorised the field, raising to prominence its conceptual underpinnings. Resilient health care, as articulated by the earlier editions of the Resilient Health Care book series edited by Hollnagel and Braithwaite alongside the late Bob Wears [2–5], emphasises the capacity of care systems to adapt and thrive amid complexity, uncertainty, and changing conditions. Unlike traditional approaches that focus on preventing errors and managing risks through compliance with standardised procedures (protective safety), resilient engineering approaches support maintaining and improving performance in real time, and in the face of unexpected challenges (productive safety). The theoretical position of Resilient Health Care is grounded in the resilience engineering concept that healthcare systems must be designed to be flexible and capable of responding to a wide range of situations, not just those that are anticipated or anticipatable; and that health professionals mostly succeed in their endeavours to keep patients safe and provide effective care. This requires an emphasis on learning, continuous adaptation, and the ability to foresee and respond to, or cope with, the unpredictable nature of healthcare environments.

All-in-all, key principles include that staff at the coalface have: the ability to monitor and respond to variability, the flexibility to adjust to both routine and unexpected situations, systemic degrees of freedom to act, and the capacity to learn from not only failures, but successes, too. The ultimate goal is to create healthcare systems that are not only robust but also capable of dynamic adaptation in the face of ever-changing demands and pressures.

The pandemic suggests that we can learn from multiple examples to observe the dimensions of health services’ and systems’ responsiveness to events and capacity of everyday clinical work to succeed in terms of resilient performance. We turn to this task.

### Aims

Our aims are twofold: (1) to present synthesised case accounts, closely analysing them to derive key themes, respectively: responsiveness; adaptiveness; local on-the-ground means of adopting policy changes; and interventional consequences of resilient performance; and (2) to distil from the cases lessons about actually-occurring health systems performance. That is to say, we want to understand the behaviours of clinical providers of care where, by virtue of their resilient performance, care is delivered in complex settings while they simultaneously keep patients safe and manage potential risks and problems on the fly. The paper asks *what do case studies of resilient performance tell us about change and its enactment over time as real-world participants go about tackling issues before*,* during or immediately after they eventuate*,* and which then never blow up into larger problems?*

## Methods

### The settings

We draw the cases from the Resilient Health Care book series [2–5], where contributors focused on the way actors within health systems improvised, adjusted, manoeuvred or performed workarounds to accomplish their aims and objectives. As we have seen, clinical actors do so in response to a desire to provide good care under challenging circumstances, striving to ensure that their systems of care continue to function. At bottom, we want to observe people in the system going about their activities, performing detailed, nuanced, everyday clinical work and adapting as necessary to presenting events and problems.

A key tenet of these books [2–5] is that everyday clinical work is by no means ordinary but is, to the contrary, remarkably accomplished. Stakeholders across healthcare settings work in ingenious ways, choreographing and coordinating their work, tasks and activities to keep patients safe. They deliver care in complex settings, succeeding in often getting things right, and ensuring that treatments, procedures and clinical services go well despite under-resourced, constrained, and often pressurised and perennially challenging environments. This is not the domain of fancy change tools, sophisticated organisational theories, or ivory-tower management academics. It is coalface participants intensively interacting with each other, solving real-world problems on the hoof, with thoughtful social researchers seeking to ethnographically document and understand the social dynamics of their care provision as they discharge their responsibilities to their patients.

### An inductive approach

Deductive case study selection is based on testing pre-existing theories or hypotheses whereby researchers start with a theoretical framework or hypothesis and select cases that will allow them to test the validity or applicability of that theory. In contrast, we took an inductive approach. In our study, case selection was driven by the aim to develop theories and categories of behaviours, and to generate new insights. We began with observations authors had made in the cases presented across the Resilient Health Care series, seeking to then identify patterns or themes that emerged by the research team in reading and re-reading the cases. We specifically looked for cases based on their potential to provide rich, detailed data that could help us identify patterns, concepts, or hypotheses to explain clinical work *in situ.* The emphasis was on exploration, allowing theories to emerge from the case-data.

### Procedures

Having read the cases multiple times, we designed and followed a three-phase procedure for selecting included cases and ultimately deriving categories that explained them.

#### Selection criteria 1

Included cases needed to be representative of front-line behaviours that reflected resilient performance.

#### Selection criteria 2

Case selection focused on illustrating retrospective analyses of disturbances or events; or provided an understanding of everyday clinical work (work as it is done in the real world in contrast to work as it is imagined e.g., in policy documents).

#### Selection criteria 3

We sought a selection of countries and case focuses represented in the Resilient Health Care books, in order to secure diversity of case topics (e.g., teamwork, technology, surge demand).

Having selected the cases, we examined them further and in detail, created a synthesis of them, and from that detailed knowledge developed four categories that emerged from our familiarisation with them. These were categories that encapsulated an overarching type of expression of resilience performance: immediacy of responsiveness; gradual adaptiveness; purposeful local adoption at the inception of a new policy measure; and interventional consequentiality i.e., the way people in the case responded to an intervention.

## Results

### The cases

The abbreviated cases are presented in Tables 1, 2, 3, 4, 5, 6, 7 and 8 below. There were eight, as follows:

- Two cases, one from Canada and another from Sweden, where the onset of the crisis was rapid, and immediate *responsiveness* was needed;
- Two cases, one from Japan and another from Belgium, the manifestations of which were gradual, and *adaptiveness* to circumstances was incremental;
- Two cases, one from the United Kingdom and another from Norway, where the resilient performance was purposefully cultivated and on-the-ground *adoption occured* in response to new policy measures; and.
- Two cases, one from the United States and another from Brazil, where in each case an intervention perturbed the system and resilient performance was an outcome, best seen *consequentially*.

### The immediacy of responsiveness

The first two cases (Tables 1 and 2) provide a window into how people at the coalface react in the face of a crisis. They show how responses can often be immediate or in close-to-real-time depending on the nature of the emergency.

**Table 1:** A crisis in an emergency department in reaction to a riot amongst Canadian hockey fans

Hunte [18] examined how the healthcare system in Vancouver, Canada, responded in a resilient fashion to a riot that broke out following the 2011 Stanley Cup. The Vancouver Canucks lost. Spectator violence in sports like hockey has been an issue for a long time, with examples documented since ancient times. Perceived injustice is a dominant explanation for such collective behaviour. In this case, heavy crowds in a small space and excessive alcohol consumption converged in fuelling behaviours that began with Canuck fans’ bottle throwing and graduated to overturning vehicles, setting fire to cars and buildings, and looting. Over a hundred injured rioters and bystanders were taken to the local Emergency Department (ED) over a four-hour period. The attendance surge was double the normal rate for ED presentations. Many participants were overcome by tear gas from the riot police, had alcohol toxicity, or mild to moderate trauma, particularly lacerations; some suffered from a combination or all of these. ED staff drew on their expertise, prior experience with other riots, and anticipatory training they had undertaken to prepare them for future possible events such as these. The staff mounted a sophisticated response to the rapidly accelerating patient load. For example, nurses quickly set up a decontamination station outside the ED to wash out tear gas, ensuring no residue made its way into the ED. They rapidly created an additional triage facility in the courtyard to immediately deal with minor injuries, while patients with more severe trauma were channelled through the existing ED triage process inside the hospital. Staff members who heard about the event or watched it on TV came in to help, even if they were off duty and not scheduled to come to work that day, which assisted with managing the increased volume of patients. Indeed, even patients chipped in to help other patients while they waited, or where they could.

**Table 2:** A crisis in Sweden arising from the 2012 Stockholm blizzard

While the ability of individuals to think creatively and adapt their behaviour in the face of a disturbance is often articulated in resilience theory, resources are necessary for resilient performance, allowing humans to “exploit opportunities to cope with disturbances” (p. 59). The authors of this case, Ekstedt and Cook, [19] describe this as *Systemic Degrees of Freedom* (*SDOFs*). Resilience is enabled in the presence of these SDOFs along with the ability of individuals to purposively exploit them. Resilience engineering—building in resilient performance—concerns the active addition of SDOFs to the clinical environment. Ekstedt and Cook discussed findings from this longitudinal study conducted in an at-home palliative care service, where the variable nature of working in patients’ homes, the severity of their illness, the number of different professionals and other informal caregivers transiting through, make this service “ripe for developing ‘gaps’ in continuity of care” (p. 62). Then, a crisis occurred in the midst of this longer-term study. An unexpected blizzard in Stockholm, Sweden in 2012 severely affected travel and access of the palliative home-care unit to 48 patients. The unit staff were anticipatory about their work in light of this disturbance, meeting as a group and rapidly agreeing to reduce or postpone the day’s nonessential work tasks so they could deal with the consequences of the crisis. Nurses were given the space and independence to re-organise care via workarounds, which involved them working and travelling by car in pairs and allowing them greater capacity to travel and access patients’ residences despite the formidable icy and snowy conditions. Familiarity with their patients and the environment facilitated this fast reconfiguration of workflows. Staff improvised and organised themselves while they were in the field, including relying on nearby family members for information and support. The pre-existing, flexibly-oriented work practices of palliative care staff were also contributory; being well-experienced with mundane intrusions in work scheduling and being loosely-coupled allowed for even greater flexibility. Staff made generous sacrifices in giving up breaks, working longer hours, and reducing administrative work in favour of more pressing clinical activities.	

This first case (Table 1) highlights the resilience of a healthcare system that was able to draw on reservoirs of collegial culture fostered at this ED over time. Staff learned from the experiences of past events; anticipated the possibility of future events including through prior training; became aware of the situation through media coverage, and undertook actions that made use of, but went beyond, the formal crisis plan. The potential for this resilient performance to emerge was not only attributable to these factors, but the nature of the event itself; a similar response might not be possible to, say, an earthquake, which could involve significant disruptions to infrastructure (e.g., technology, resources, transport), and an even greater accelerated increase in the influx of ED patients, or injuries to off-duty staff themselves. Early in the riot, clinical work in progress was quickly completed before new riot-induced clinical workloads swamped the ED, which enabled the continuous resilient performance of the staff despite the onset of the crisis.

In the second case (Table 2), the unit staff exhibited resilient performance in response to the disturbance of the blizzard, anticipating, planning on-the-fly, and adapting to the trying circumstances they encountered. The authors of the case indicated that these novel work expressions were made possible by pre-existing flexibility inherent in the system, which had been developed through investments in cultural capital that created the SDOFs in the first place. For example, the palliative care unit staff were well-versed in adapting their behaviour to the perennially variable work inherent in at-home care. Personnel were adept in behaving autonomously and they and their work were relatively loosely-coupled [26]. Some of these prior investments in resilience had matured more slowly (e.g., a professional culture of autonomy was earned and built over time) and others more rapidly (e.g., instituting morning meetings to share information). These are recognised approaches to more resilient performance [6]. This case highlights that resilience is often expressed as a trade-off: requiring the sacrifice of one goal in the interest of achieving a more important goal (ignoring less pressing tasks such as administrative procedures in favour of key clinical tasks, for example). Responsiveness to urgent problems in such cases is thereby enhanced.

### Gradual adaptiveness

We turn to considering change that occurs more incrementally, often exhibiting the characteristic in complex systems theory known as emergence. Two cases exemplify this phenomenon: one from Japan and one from Belgium (Tables 3 and 4).

**Table 3:** The gradual adaptation of responses to safe handling of potassium chloride in Japan

The complex nature of healthcare delivery often requires adaptation rather than the adoption of strict control measures. Instead of trying to micro-manage clinical work, encouraging things to go right and understanding the “what” and “how” of everyday clinical work are important considerations. We can differentiate here the concepts of work-as-imagined (WAI) and work-as-done (WAD). WAI can be represented by the policymaker who might specify in great detail a complex safety procedure and how it should be carried out. WAD is the actual, much messier, way the procedure is executed, in the context of a host of situational variables. One distinction between WAI and WAD with implications for patient safety concerns is the handling of potassium chloride (KCl), a potentially-fatal solution when administered rapidly or in the wrong concentration. Hence, WAI protocols for safe handling of KCl exist that emphasise removal of ampoules of KCl in ward storage for non-critical care and use of pre-mixed solutions (reducing potential for rapid injection), restrictions on ampoules storage in critical care, strict safety procedures and staff education. In this case from Japan, increasingly cautious KCl alerts were introduced over time because early ones were still followed by fatal events. One of these alerts (use of prefilled syringes connectable only to bags or bottles, hence mandating dilution) impinged upon the ability to administer concentrated KCl when required. This alert was believed to have led to workarounds (e.g., drawing from a prefilled syringe designed for dilution into another to produce a concentrated solution). This suggested misalignment of WAI and WAD in KCl handling. In presenting the case, Nakajima and Kitamura [20] sought to better understand WAD by collecting data on the rates of nationwide compliance (from accreditation survey data with the Japan Council for Quality Health Care (JCQHC)). They examined reasons for barriers to compliance with the recommendation of removal of KCl ampoules from patient care areas with data extracted from minutes of committee meetings and interviews with clinicians collected from one critical-care hospital. The researchers were also interested in adjustments in handling KCl in response to JCQHC recommendations, assembling data from observations of medication practices, medication supply records, and interviews nationally with physicians and patient safety officers. In terms of compliance, 170 of 400 hospitals were still stocking concentrated KCl in patient care areas, citing necessity, although many had switched to use of prefilled syringes that prevented rapid injection. Interviews with physicians and meeting minutes of Osaka University Hospital identified two major reasons for stocking ampoules in wards. One: treatment of specific patients (with hypokalaemia, i.e., severe heart failure) requires strict volume control, and hypokalaemia must be quickly treated because of the potential for life-threatening arrhythmia. Two: storage away from the area (in the pharmacy department as mandated) would delay immediate delivery of KCl. Hence, a paradox was uncovered: strictly following safety guidelines would potentially come at the expense of patient lives. In addition, adjustments on-the-ground involved stocking ampoule-like products in ICUs, against formal recommendations, or drawing the concentrated solutions from the prefilled syringes, a practice that received varying levels of acceptance across hospitals. All-in-all, there were clear differences in WAI, which had been designed to stop the rare, but fatal occurrence of rapid KCl injection, and WAD, in which the everyday practice of saving patient lives in ICU might be dependent upon rapid and immediate access to concentrated KCl.

**Table 4:** The adoption of technology within a socio-technical system in Belgium

Through a number of studies, Nyssen and Blavier [21] examined changes, flexibility and resilience of the socio-technical system in Belgium in the face of a newly-introduced surgical technique. In comparing standard laparoscopic and robotic surgery for digestive and urological conditions, they demonstrated that robotic surgery demanded more resources from a surgical team, with the procedure taking longer and necessitating greater levels of communication among the team; their research suggested a breakdown in team collaboration. At the same time, the increased communication needed, and slower pace of completing the robotic procedure, were evidence of adaptation to changed circumstances by the team. While new technology is often introduced in healthcare with the intention of making patients safer, as Nyssen and Blavier indicate, it requires medical and associated staff to adapt their practices in order to integrate the technology while maintaining a level of performance and safety. Such has been the case with the advent of robotic surgery, which is intended to supplant standard laparoscopy. Team members displayed resilience in response to the new technology. Indeed, some surgical situations necessitated robotic surgery be abandoned in favour of a back-to-the-future return to more conventional strategies (laparoscopic or open surgery). Thus, resilience was evident in the strategic flexibility of experts to the demands of the task in being willing to return to prior methods.	

Nakijima and Kitamura’s case (Table 3) shows how two patterns of collective behaviour—stock the ampoules, stock the prefilled syringes only and adjust to draw from them—can emerge from a complex system in response to the JCQHC recommendation. These behaviours were guided by simple rules (be safe, save lives). While the WAD adjustment was functional for staff delivering care to patients, it poses safety risks (e.g., mix-up with other medications, rapid injection). This led to another WAI alert about not drawing from syringes, which may itself cause other WAD adjustments to emerge. The gradual adaptiveness and dynamic interplay between what policymakers in WAI mode are trying to achieve, and what clinicians in WAD mode are trying to achieve, can often lead to contradictory or counter-intuitive responses, including new problems.

In further work reported by Nyssen and Blavier in their chapter (Table 4), a cognitive experimentation study suggested the limits of adaptive expertise to new technology: while individuals trained with laparoscopic techniques were able to recover a similar level of performance when using the robotic instrument, the same was not true of those originally trained using the robotic system who attempted laparoscopic systems. This finding, and the findings from the synthesis of the case in Table 4, suggests there are limits to cognitive flexibility and that there is the potential for creating rigidity through the uptake of new technology, which can be countered by new levels of resilience, in this case by converting back to laparoscopic surgery when the surgical situation demanded it. This research raises questions about how and when to train surgeons on new and older surgical techniques, and whether they require ongoing practise. But for our purposes, the case and associated research by Nyssen and Blavier [21] illustrates how resilient performance manifests over time as one circumstance after another emerges in a constant dance of events and decisions.

### Purposeful local adoption at the inception of new policy measures

Changing the focus again, the cases in Tables 5 and 6 provide examples of front-line actors having to localise their plans and management to take account of top-down, nationally-mandated policy initiatives. One is the introduction of a new policy affecting EDs in the English National Health Service (NHS); the other, a change in policy affecting transitioning patients in Norway.

**Table 5:** The introduction of a new policy and targets for the handover of UK patients in an emergency department

Sujan and colleagues [22] examined differences between WAI and WAD during the handover of patients at an ED. They explored how informal practices of paramedics during handover constituted a mechanism of resilience. Handover involves the transfer of information and professional responsibility for a patient from one health professional, team, or department, to another. In the ED, this process is complicated by the different disciplines involved and the professional demands on the paramedics and nurses who typically participate in the handover. A new national policy and targets were designed to improve patient safety by reducing the time ambulances were at the hospital. This was contrasted with the importance of ambulances being out in the community, and available to provide care. The policy involved the designation of one nurse coordinator who would receive all handovers from paramedics for ED patients arriving by ambulance and record them via a formal tool, ensuring a consistent transfer of relevant information. However, this WAI initiative was not always carried out in practice. Sujan and colleagues found that paramedics were involved in a ‘secret second handover’ in which further information was relayed to an ED nurse who would take over care for the patient. The ED nurse would typically be appreciative of the extra detail provided beyond the formal information transferred as dictated by the tool. A tension arose in the goals between the nurse coordinator who, taking a managerial perspective, considered the second handover an unnecessary duplication which might diminish the ability to meet handover targets, and the paramedics who had a strong sense of personal accountability for their patient and would linger in the ED, trying to ensure the informal transfer of other information they deemed necessary, particularly when the case seemed to call for it. Lingering, of course, had consequences as the ambulance would not be back on the road, available for other patients in the community. As such, paramedics had to negotiate between their responsibility to their current ED patient and other potentially ill individuals in the community requiring an ambulance.

**Table 6:** A policy reform measure and its effects in two Norwegian hospitals

In the early 2000s in Norway, a reform was implemented to encourage the transition of elderly patients being discharged from hospitals into follow-up primary care institutions organised by local municipalities. This reform placed financial burden on municipality services for the transitions, as well as requiring legal agreements between hospitals and these services, including specifying what information to exchange and who was responsible for which component of the transition. There were stringent deadlines imposed for discharge planning. Through an ethnographic study, the researchers Laugaland and Aase [23] examined the patterns of discharge in three hospital wards (geriatric, medical, surgical) in two hospitals. Laugaland and Aase identified three main ways the services adapted to the reform measures. The first involved increased discharge planning between hospital and primary care that led to improved dialogue between the providers. These additional tasks simultaneously imposed a greater administrative burden on participants (e.g., primary care staff making early contact with hospitals to further assess patients’ conditions and post-discharge care needs). The second was increased flexibility in primary care services to receive patients, with services negotiating with other nursing homes for beds, with the hospital to delay discharge, or working around shortages by moving patients directly to at-home nursing care. Alongside this flexibility, though, was prioritisation of the needs of patients being discharged over individuals at home who also required a short-term stay in a nursing home, and an increased number of care transitions that might not be conducive to healing elderly patients (e.g., a patient pathway might involve moving between the hospital to an interim nursing home ward to a rehabilitation ward and back to hospital). Third, time-efficiency on the day of discharge was greatly enhanced, with primary care services responding rapidly with an assigned bed when a patient in hospital was ruled medically-fit and ready for discharge. This in turn required increased efficiency among hospital staff to plan discharge; however, it was notable that the time of day affected the determination of ‘medically-fit’ and this new process of discharging patients.	

The case study in Table 5 demonstrates the ways in which formal WAI instructions do not always align with informal WAD arrangements due to the demands, targets, personal responsibilities and tensions inherent in patient care roles. While a secret second handover might be viewed from a managerialist, WAI viewpoint as a wasteful practice that diminishes the ability to meet government-mandated targets for rapid handovers, it might alternatively be a key plank in local management and planning to ensure the quality of patient care, but balanced against the expense of other potential patients out in the community. Those working in emergency medicine were adapting their behaviour, displaying resilience in the practice of the secret second handover, trading off the need to be formally meeting targets with enacting quality patient care as the conditions required it, and as they saw fit.

While the Norwegian reform measure was considered effective (Table 6), the nuanced nature of the outcomes, particularly for the primary care services, demonstrates that a key tenet of resilience theory—that staff produce acceptable outcomes under varying conditions—requires recognition of the multiple perspectives (healthcare provider, clinician, patient) through which those outcomes may be evaluated positively or negatively. Reforms of this kind are inherently complex; in this case there are two systems (hospital and primary care) interacting, increasing the complex manoeuvring, negotiating and trade-offs of the parties. Recognition of the boundaries between the two is important because a positive outcome in one system may lead to less successful outcomes or even demonstrably poorer effects upon the other. Recognising the dynamic nature of this system over time also requires thoughtful consideration by staff because while this particular reform met with its stated aim, it may have had, in some circumstances, a number of less positive downstream effects (e.g., increased care transitions and returns to hospitals). In the end, though, it was incumbent on staff to plan and manage the way the policy reform measure was taken up locally. In complex systems, resilience and adaptation in the light of top-down reform needs to be responded to in adroit, considered ways by those on-the-ground.

### Interventional consequentiality

We now reflect on the way staff in complex health settings respond to interventions (Tables 7 and 8). There are multiple downstream effects of any intervention. In this final couplet, we draw on one case from the United States and a second from Brazil.

**Table 7:** An intervention using simulation to enhance resilient clinical practices in the United States of America

Deutsch and colleagues [24] considered how simulation can be used as a tool for investigating resilient healthcare performance. Simulation involves the use of realistic, engaging scenarios for the purposes of learning and training, and is useful in a complex and dangerous environment like healthcare where, as the authors note, performing via simulation before practicing in the real world on actual patients can reduce risk. In this case a simulation study team was brought in to assist with the opening of a new paediatric community hospital. The study team’s role was to assess the new clinical staff and facilities, identify and mitigate latent safety threats (LSTs), and develop some potential scenarios the staff were likely to encounter, which could then be simulated. The mixed-methods study of simulation incorporated a set of design features including reinforcing appropriate actions and resources, making constraints visible, and developing team behaviour and adaptive capacity. There were two stages. First, eight centre-based simulations were used to define the roles and scope of practice of the actors; in the second stage, sixteen in situ simulations at the hospital facility were conducted, intended to assess the quality of initial solutions and identify any unintended consequences. Unique multidisciplinary teams participated in the same scenario. These simulations were filmed and scored for team behaviours by reviewers, allowing for comparison between different conditions and teams. Further data collection involved staff completing a workload scale immediately post-simulation (used to assess the limits of safe performance) and participating in audio-recorded debriefing, including a video review of the simulation. Staff not participating in one simulation were able to observe the video and contribute to the debriefing. Following these sessions, staff filled in an electronic survey (capturing perceptions staff may have otherwise felt uncomfortable providing, or new reflections). The staged design of the research allowed for examination of how the patterns of behaviour and teamwork changed in light of the first stage results, changes in the environment, and team configuration. Results for the centre-based simulations highlighted a number of themes and LSTs (e.g., that clinical leaders should endeavour to only lead, and not become involved in providing care or conducting procedures; and knowledge gaps related to weight-based dosing). Scale scores also indicated that medication nurses had the highest workload, something that in situ simulation demonstrated was partly due to the configuration of the workplace. Overall, the study identified 37 LSTs related to equipment, medication, personnel, and resource issues, in addition to other issues of cognitive fixation and loss of situational awareness during simulation, and in some cases poor teamwork. These challenges could now be addressed ahead of the opening of the hospital.

**Table 8:** Introducing process improvements in a university hospital in Brazil

Saurin and colleagues [25] examined how process improvements (e.g., making healthcare more lean by stripping out redundancy), which are now ubiquitous in many healthcare settings due to rising pressures on the system, affect resilient performance. They investigated the process of redesigning the preparation and administration of drugs at a university hospital in Brazil using lean and process improvement tools. A unit was chosen to be the pilot of the study, because it was similar to another unit in the hospital (allowing for replication), complex (i.e., socio-technical, diverse range of drugs administered), and where staff were committed to safety (i.e., as exemplified by a high number of safety reports). The authors utilised a Design Science Research methodology—a means of solving a practical problem through creation of an artefact (i.e., an intervention framework integrating resilience principles and lean improvement)—while recognising the system they were intervening in, which would prohibit a prespecified, perfectly predictive solution devised at the outset. Staff participated in data collection and analysis, contributing their understanding and knowledge of problems they faced. A mixed-composition design team was intended to ensure the final artefact was appropriate to the work and workspace, while not producing simplistic linear solutions. The research team collected both quantitative data (from time and motion studies) and qualitative data (from observation, document analysis, a focus group) from different perspectives, intended to understand WAD, while describing the system with reference to both lean and resilience principles. The lean approach was used to map the processes (i.e., processing, waiting, transporting, inspecting) related to a service or product (i.e., preparing and administering drugs), and used quantitative data (e.g., lead times, work-in-progress). The resilient lens, alternatively, provided a deeper understanding of WAD, such as how variability occurs and can resonate through the system, by characterising the relationship between activities (i.e., input, output, time, control, preconditions and resources). Data collection concluded with discussion of the analysis with staff at a focus group. Documents were analysed using content analysis looking for stages (the lean lens) or functions (the resilient lens), clients and their needs, and the problem according to process stages or functions, and possible solutions. The lean lens identified 19 process stages and six clients (e.g., patients, nurses); clients had 41 needs associated with 45 problems. The resilient study identified 16 functions, with 12 having substantial variability. While lean and resilient approaches can often correspond, the resilient approach reportedly provided greater detail and nuance because it did not model a linear relationship between stages (e.g., administration of drug interacts with six other functions). Problems (e.g., medications not labelled according to patient when coming from pharmacy) were analysed using six guidelines for socio-technical systems (give visibility to process and outcome, anticipate/monitor the impacts of small changes, encourage diversity of perspective, design ‘slack’, monitor/understand the gap between prescription and practice, create an environment supporting resilience).	

The Table 7 findings suggest that the intervention of a simulation provides an opportunity to understand WAD in the context of a complex socio-technical system. The study demonstrated resilience principles: monitoring (i.e., participants learnt to observe patient conditions and the interactions among other team members, such as recognising that the physician leader was preoccupied with clinical tasks, diminishing his or her ability to lead); responding (e.g., adjusting the resuscitation team’s structure to provide more assistance to medication nurse); learning (e.g., practising technical skills); and anticipating (e.g., the research team working to predict common scenarios clinical staff would encounter and creating simulations of them). The outcome was better-prepared staff for subsequent workplace challenges.

Solutions to problems identified as a result of the Table 8 intervention were assessed using resilience principles. A number of remedial strategies resulted, such as delivering drugs from pharmacy with a patient tag and creating a help chain. The resilient approach facilitated understanding of the likely effect of changes on other activities and functions of the system (e.g., to reduce variability). Overall, the analysis highlighted how components of the system adapted to varying extents (e.g., management adapted to new drugs by ordering them, but those preparing and administering drugs did not adapt to new administration requirements). Depending on the circumstances, such changes could contribute to a brittle system. As the Brazilian authors note, these are important issues to consider: healthcare systems evolve constantly, and increasingly through the introduction of formal improvement processes, i.e., interventions; resilience engineering helps understand why certain countermeasures, or solutions, do or do not make sense in a given context, downstream from the intervention.

## Discussion

Having presented the cases and commenting briefly on each *individually*, we now move to a discussion of what we have learnt about in situ resilient performance under varying conditions *across* the cases. Table 9 provides summary information on the health systems in the study and a short digest of each case. This is followed by a discussion bringing our observations together, the consequences for change, and the implications of this work across time.

**Table 9:** The health system characteristics, case settings, type and core issue addressed

Case	Country	Life expectancy	% GDP on health	Type of health system	Case setting and institutional orientation	Case type	Core issue
Nyssen and Blavier [21]	Belgium	82.17	10.3	Mandatory through state social security scheme, with a complementary system funded through private health insurance	Operating theatres – hospital setting	Adaptiveness	Communication and use of technology
Hunte [18]	Canada	82.96	11.5	Publicly funded single payer healthcare system, with a complementary private sector	Emergency department – hospital setting	Responsiveness	Coping with surge in demand
Laugaland and Aase [23]	Norway	82.94	10.4	Universal health coverage through taxes and payroll contributions, small private system of health clinics	Transitions between hospital and primary care	Planning	Working across systems
Ekstedt and Cook [19]	Sweden	83.33	11.9	Taxation-based public system with small private system	Home care – community setting	Responsiveness	Delivering safe home care
Sujan, Spurgeon [22]	England	81.77^a^	10.2^a^	Publicly funded single payer system with contracting to some private providers	Emergency department – hospital setting	Planning	Communication during handover
Deutsch, Fairbanks [24]	US	79.11	17.9	Government programs and private health insurance funded through employers, but not mandatory	Simulation of care – hospital setting	Consequentiality	Understanding teamwork and work-as-done
Nakajima and Kitamura [20]	Japan	85.03	10.9	Mandatory health insurance either through employer or national program	Intensive Care Unit – hospital setting	Adaptiveness	Safety and workarounds in medication administration
Saurin, Rosso [25]	Brazil	76.57	9.6	Taxation-based free and universal public system	Wards – hospital setting	Consequentiality	Variability in medication administration

^a^United Kingdom data

### The cases in context

Analysing eight cases assigned to four categories of change and improvement in resilient practices has offered insights into care delivery. It has also brought to the foreground ways in which actors, particularly those on the front lines of care, engage with improvement activities of their own, or those interventions stimulated by translational research teams or other external interventional mechanisms, e.g., nationally-mandated reforms.

Authors of these case studies have played prominent roles in not only describing responsiveness to change and the capacity of people on-the-run to provide good care continuously (e.g., Hunte’s Canadian riot case and Nyssen and Blavier’s Belgian technology adoption case) but actively participating in improvement activities (e.g., Deutsch, Fairbanks and Patterson’s US simulation study and Saurin and colleagues’ Brazilian process improvement case). When change is incremental, researchers inevitably need to spend more time in situ understanding what is going on in order to thoroughly document the context, circumstances and events as they unfold [22, 23].

But when change has rapid onset, we can see people reacting very swiftly and effectively, with patient care seen as an imperative, with other tasks or activities being de-prioritised. In regard to reactions to crisis in the Canadian and Swedish investigations, for example, we saw skilled improvisation of ED staff on the one hand and palliative care professionals on the other, responding purposefully to the challenges that a riot and a blizzard respectively posed to patients, and putting aside non-essential tasks. Facing accelerating pressure and considerable risk of disabling disruption, the response was to maintain care quality, de-prioritising extraneous tasks (e.g., documentation) in favour of immediacy and necessity (e.g., prioritising direct treatment and communication with patients, families and colleagues). Staff were experienced, prepared, capable and confident to do what needed to be done.

We also observed the adept adaptation and adoption, and the flexibility and ingenuity required by care teams to embrace thoughtful change, exemplified by the Belgian robotic surgery case and the Japanese potassium chloride case. Staff in these studies accepted the challenges they faced, and displayed manoeuvrability over time through learning from practice, and taking on board what works, rejecting what doesn’t, and building the new practices successively, reflecting on progress after each incremental change.

When we get to the cases in Tables 5 and 6, we can see situations where change is tailored to the specific setting, and managed and planned locally, following bigger-picture policy change mandated nationally. Laugaland and Aase documented, in their case, the responsiveness to reforms of care transitions and discharge in Norway and the changes to practices necessitating on-the-ground adoptive flexibility while mobilising additional or alternative available resources (e.g., using nursing homes as an interim staging point between hospital and home). Sujan and colleagues by way of contrast, but with echoes of the Laugaland and Aase case, showed how informality and the mobilisation of capacity to make things work better in informal ways via the secret second handover resulted in better care for patients. Despite tightly-defined policies, in both cases personnel at the sharp end exhibited high degrees of manoeuvrability and multiple levels of improvisation—and by their actions, mitigated against future problems. Instead of slavishly following mandates, front-line personnel exhibited reflexivity and thoughtful reflection on what to do in their responses. This is reminiscent of the famous literature on street level bureaucracy [27, 28] whereby people with direct contact with the public can exercise considerable discretion and can employ degrees of agency—they are, when acting at the top of their scope, mediators and interpreters of policy and guidelines rather than subservient enforcers of it. Bottom-up responses often reflect localised nuance and contextual implementation, as exhibited in these cases.

Investigating how specific interventions are handled was the province of the cases in Tables 7 and 8. The Brazilian and US cases illuminated how interventional, action-orientated research can change behaviours, strengthen preparedness, and enable good outcomes, acting to enhance resilient performance. An important point of emphasis in those cases is that such results can endure long after the intervention itself has ended.

### Lessons for change

All-in-all, everyone at the sharp end of healthcare delivery knows that they can only accomplish their work if they adjust what they do to meet the circumstances they face. Indeed, for any system to succeed and be resilient is to be able to respond, to monitor, to learn and anticipate [29]. Riven through the pages of these case descriptions are these resilient activities. The signature watchwords of resilience engineering can be seen spontaneously emerging in a myriad of ways. These attributes of successful front-line performance are the outworking of people who are essentially muddling through with purpose—finding solutions as they go along, making the imperfect system work, figuring out the means by which goals can be achieved, sifting through complexity and sorting issues out before they accelerate or degrade, regardless of constraints, pressures, or poor systems [7]. 

Our message, in the end, is a plea: that we should not rush to manage change, or introduce new tools, frameworks or policies into healthcare just because a new fad arises or everyone else seems to be doing something different. The next big thing is always just around the corner in healthcare. Beware when the the freshly-released tool, shiny checklist, novel framework or irresistible new policy comes along.

Instead, our take-home from these cases is to look for inspiration in the extraordinary accomplishments of everyday actions that get things right far more than they get things wrong, and to the way people, mostly unseen, spend time fixing things many of which never worsened or blew up, because the front-line staff resolved them before they became a problem or as they emerged. Witness the long-term persistence of the Japanese workarounds in medication administration, the resourcefulness of the second secret handover, the caring responsiveness to blizzards and riots, and the embedded ingenuity inherent in the other case participants.

## Conclusions

Attempting to deeply understand everyday coalface work is a lesson in humility. There are no magic wands or silver bullets, and change models and tools offered up in glossy trade journals or consultants’ websites are typically not much more than labels looking to attach themselves to an ardent believer. We prefer examining how WAD occurs as it emerges, in all its variability, ingenuity, resourcefulness and even grandeur—although many people treat ordinary work as banal. What we see when we do this is intelligent and resourceful front-line staff solving important problems, largely incognito. Healthcare is far more about the unsung heroes than the new-fangled managerial tool, PowerPoint-drawn change model or recycled social theory. Let’s do more studies of people in situ, and see them doing their thing, with less interference from high level policymakers, enriched consultants and overly inventive management theorists armed with a best seller management book purporting to have all the solutions to healthcare’s ills. It turns out the people who count in healthcare are the ones in the trenches, every day, working round the clock, offering care, 24/7. Health systems must therefore continuously support and nurture front-line healthcare workforce capacity to maintain resilience.

## Data Availability

All data generated or analysed in this paper are included in the following published books, which are all part of the Resilient Health Care series: R. L. Wears, E. Hollnagel and J. Braithwaite (eds), Resilient Health Care Volume 2: The Resilience of Everyday Clinical Work; E. Hollnagel, J. Braithwaite and R. L. Wears (eds), Delivering Resilient Health Care; and E. Hollnagel, J. Braithwaite and R. L. Wears (eds), Resilient Health Care. Each case study is drawn from a chapter in one of these books.

## Abbreviations

ED: Emergency Department
SDOFs: Systemic degrees of freedom
WAI: Work-as-imagined
WAD: Work-as-done
KCl: Potassium chloride
JCQHC: Japan Council for Quality Health Care
NHS: National Health Service
LSTs: Latent safety threats
